# Knowledge and attitude of women regarding breast cancer screening tests in Eastern Iran

**DOI:** 10.3332/ecancer.2018.806

**Published:** 2018-02-05

**Authors:** Azra Izanloo, Kamran Ghaffarzadehgan, Fahimeh Khoshroo, Maryam Erfani Haghiri, Sara Izanloo, Mohadeseh Samiee, Alireza Tabatabaei, Azadeh Mirshahi, Morteza Fakoor, Najmeh Jafari Moghadam, Sayyed Majid Sadrzadeh

**Affiliations:** 1 Razavi Cancer Research Center, Razavi Hospital, Imam Reza International University, Mashhad 91735-1453, Iran; 2 Islamic Azad University, Branch of Trobat, Trobat, Iran; 3 Department of Radiology Technology, School of Paramedical Sciences, Mashhad University of Medical Sciences, Mashhad, Iran; 4 Department of Emergency Medicine, Faculty of Medicine, Mashhad University of Medical Science, Mashhad, Iran

**Keywords:** screening, early detection of cancer, knowledge, breast cancer, East of Iran

## Abstract

**Introduction:**

According to recent statistics, there has been a rapid growth of breast cancer in developing countries. Thus, early detection is essential. This study is based on the perception of people in the Northeast of Iran regarding breast cancer screening.

**Methods:**

In a cross-sectional study, 1469 women were selected randomly in the period from April to November 2016. The study population consisted of women or their companions referring to outpatient clinics or people in public urban areas who filled out a breast cancer screening questionnaire in an interview.

**Results:**

The patients’ age was in the range of 14 to 84 years (mean = 38.8). More than 84% of interviewees were not informed of breast cancer and screening tests. The main reasons mentioned by patients for their failure to do screening tests was ‘absence of any symptom or problem’ and ‘they did not think it was necessary’.

There was not a significant difference between income level, marital status and knowledge of people about breast cancer screening tests (P > 0.05). However, employment, education level and family history had a positive effect on people’s awareness of breast cancer and its screening tests (P < 0.05).

**Conclusion:**

The lack of knowledge in people from low socio-economic classes was the main barrier to breast cancer screening. In this regard, organizing training programs by physicians and the media can help raise screening rates.

## Introduction

Breast cancer is the most prevalent type of cancer among women in Iran and other parts of the world [1]. It is still a major health problem in both developing and developed countries. According to recent statistics, breast cancer is rapidly increasing in developing countries [2]. In Iran, breast cancer accounts for 76% of all common female cancers [1]. It is the fifth major cause of cancer-related death in Iranian women after gastric cancer, leukaemia, lung and bronchus cancer, liver cancer and cholangiocarcinoma, which take the lives of 1,200 women each year (with a prevalence rate of 3.4 per hundred thousand women) [3]. In a 2000 study, 70% of breast cancer cases in Iran were diagnosed at Stage III [4]. The results of another 2006 study revealed that after raising awareness and knowledge of patients, 60% of cases were diagnosed at Stage II [5].

Some studies have shown that raising awareness of women and modifying their behaviour can precipitate the stage of disease detection and therefore change the quality of life and survival rate of patients (which are directly related to the stage of cancer detection).

Several studies have revealed that the age of breast cancer diagnosis in Iran, which is nearly 10 years younger than other countries, is between 40 and 50 years [1, 4]. In developing countries such as Iran, self-assessment is an appropriate selective screening test [6]. Knowledge, awareness and societal attitudes are the most important factors for successful implementation of any screening program. Given the ethnic and cultural dispersion and demographic features of Iranian people, and the absence of a national breast cancer screening program, this study was undertaken on a large study population in one of the metropolises of Iran to explore women’s awareness and attitude about breast cancer and barriers related to screening tests.

## Method

This is a cross-sectional study in which 1,469 patients referring to outpatient clinics and people in public urban areas, who were aged above 14, were interviewed and asked to fill out the study questionnaire in the period from April to November 2016.

Interviews were performed by four trained questioners. Questionnaires consisted of demographic data, knowledge of symptoms, risk factors and screening tests for breast cancer as well as the reasons for failing to do these tests. The ways in which participants learned about screening tests were also investigated.

Risk factors of breast cancer assessed in the questionnaire, which were based on the NCC 2016 guidelines, consisted of 17 items: (1) excessive alcohol consumption; (2) smoking; (3) obesity; (4) family history and genetics; (5) chest radiation before the age of 30; (6) sedentary life; (7) number of pregnancies; (8) pregnancy age; (9) number of breastfeeding; (10) breastfeeding age; (11) high-fat diet; (12) dense breast; (13) menstrual age; (14) contraceptive use (15) family history; (16) patient age and (17) hormone therapy [7].

Symptoms of breast cancer included six items: (1) painless mass; (2) premenstrual breast nodules; (3) sharp pain in the nipple; (4) breast asymmetry; (5) galactorrhea after breastfeeding and (6) blood discharge from the nipple [7].

In our study, women’s knowledge of breast cancer screening tests was also evaluated: (1) self-assessment; (2) ultrasonic; (3) mammography and (4) MRI.

Since the purpose of the study was to evaluate women’s knowledge of breast cancer screening tests, patients with a history of breast cancer or related diseases were excluded. After interviews, free training about the use of screening tests was offered to participants.

The reliability of the questionnaire was evaluated by Cronbach’s alpha (a = 86.4%). The validity of the questionnaire was assessed using constructs of health belief model as a guide.

Descriptive and analytical statistical analyses were performed by SPSS Statistics V21. The level of awareness was compared across the level of education, marital status, income and employment using Chi-square and ANOVA for quantitative data such as age and knowledge. The scoring of different sections of the questionnaire was conducted as shown in Table 1.

## Results

About 1,469 questionnaires were completed by women in the age range of 14–82 years (mean = 38.8, SD = 11.69). Demographic information of patients is given in Table 2. At the time of this study, most patients (82.6%) were married.

In most patients with a family history, breast cancer had already appeared in a relative such as an aunt (2.9%) or the mother (2.6%). Overall, more than 84% were not well informed about breast cancer and its screening tests. Among these, as shown in Table 3, more than 54% were unaware of the risk factors of this cancer. The greatest awareness was observed in women who were able to identify two or three risk factors. Table 3 shows women’s knowledge about breast cancer and screening tests. Most women cited self-assessment (41.6%) and ultrasound (46.4%) as the main screening methods. Among six major symptoms of breast cancer, most patients (47.9%) referred to a painless lump as a symptom of cancer.

More than 26% of participants stated that their failure to do screening tests was mainly rooted in ‘the absence of any symptom or problem’ and 17.6% said that ‘they did not think it was necessary’. A large number of participants (29.1%) believed that doctors could be especially helpful in informing the community about screening tests for breast cancer and 24.1% stated that the Internet and social networks were a good means of raising public awareness. 19.3% of women also contended that mass media could play a significant role in this regard. Data analysis showed a significant relationship between variables such as family history of breast cancer (*p* = 0.029), working status of women (*p* = 0.0001), educational level (*p* = 0.0001) and women’s awareness of breast cancer risk factors. The results showed that employment and level of education had a positive effect on knowledge and awareness of women about breast cancer and its screening tests. Moreover, the results indicated a significant disparity between income status (*p* = 0.972), marital status (*p* = 0.808) and women’s knowledge of breast cancer and screening tests.

The results showed that women’s knowledge of risk factors (*p* = 0.165) and symptoms of breast cancer (*p* = 0.248) were not significantly correlated with age, but older women were more aware of the type of screening tests for breast cancer. Thus, this difference was significant at different ages (*p* = 0.009).

## Discussion

The study found that more than 84% of the participants were not well aware of risk factors, symptoms or screening tests for breast cancer, and more than 59% had never taken any screening tests before. This figure is even lower than the one reported in the United States, according to which about 72.6% of women aged 50–74 years have breast cancer mammography screening tests done every two years, though it is still below the target rate [8]. Also, 73% of Caucasian women and 72% of Asian women aged 50–74 years have mammography tests performed on a yearly basis [8]. The results of the present study are consistent with the findings of another study according to which 34% to 65% of Korean women residing in the United States went for breast cancer screening tests [9–12].

This study revealed that one of the main challenges facing the use of screening tests was a lack of knowledge about the necessity and importance of doing these tests, with few people pointing to the costs of tests as an impeding factor. However, Pourfarzi *et al* [13] reported that the high cost of breast cancer screening tests was a major factor discouraging people from the administration of these tests, which is in agreement with studies like Farid *et al* [14] and Shamsi *et al* [15].

A study in Saudi Arabia suggested that despite subsidies allocated to screening tests, so that women could access these tests free of charge, they were not well received by women [16]. These contradictory results have urged some researchers to conclude that cultural beliefs of people also affect their tendency to present for screening tests, urging Lana Sue to consider patients’ awareness as one of the leading factors in breast cancer screening [17].

In the same line of research, Crawford stated that social and cultural demographic factors should also be considered in planning relevant interventions [18]. It has been reported that each year more than a million and a half women are diagnosed with breast cancer and 502,000 patients lose their life because of this disease. Of these, nearly 60% of the deaths from breast cancer occur in less developed countries (despite it being seen as a disease of the developed world).

As such, greater attention should be paid to breast cancer prevention and screening. In this regard, widespread propagation and raising public awareness about screening tests should be high on the agenda of healthcare systems. The results of this study, consistent with the literature, suggest that informing people through the media and physicians, as the most reliable sources of information, can be very helpful [19].

One limitation of the present study was that subjects filling out the questionnaire were chosen from both the hospital environment and public places, and those in the hospital were often more aware of breast cancer than were the other subjects, which can cause heterogeneity in the study population. On the other hand, since questionnaires were completed by questioners and they provided a varied extent of explanation to participants, it may have caused bias. It is suggested that other studies be carried out in light of the above factors.

## Conclusion

Lack of knowledge, especially in the low socio-economic stratas of society, is one of the most important barriers to screening tests. Therefore, organising training programs with the cooperation of doctors and the media is crucial to improve the uptake of screening tests.

In a relatively developed country like Iran with a solid health care system, the training offered by family doctors could be a helpful strategy for promoting breast cancer screening based on national and international guidelines for ultrasound, and if necessary, mammography tests.

## Figures and Tables

**Table 1.** Scoring of different parts of questionnaire.

Scoring	Part
Lack of information = nothing Aware of 1–5 items = low Aware of 6–10 items = medium Aware of 11–17 items = high	Awareness of the risk factors of breast cancer
Lack of information = nothing Aware of 1–2 items = low Aware of 3–4 items = medium Aware of 5–6 items = high	Awareness of the symptoms of breast cancer
Lack of information = nothing Aware of 1 item = low Aware of 2–3 items = medium Aware of 4 items = high	Awareness of the screening tests

**Table 2.** Demographic characteristics of the study participant.

Variable	No.	%
Marital status Single Married	221 1214	15.10 82.9
Widowed/divorced	28	2
Education Diploma or less Some college or Bachelor’s degree Master’s degree or more	670 683 114	45.8 46.5 7.7
Employment status Employed or retired Unemployed	484 983	32.9 67.1
Monthly income (US dollars) 270> 270<	660 735	47.3 52.7
Family history of breast cancer Yes No	197 1,265	15.5 86.5
Health insurance Yes No	876 588	59.8 40.2
Prior history of breast cancer screening Yes No	591 874	40.4 59.6

**Table 3.** Distribution of knowledge score about risk factors and symptoms of breast cancer and screening tests.

	Knowledge of symptoms	Knowledge of risk factors	Knowledge of screening tests
High	5%	2.2%	19.5%
Medium	11.3%	8.2%	17.2%
Low	41.5%	34.5%	25.8%
Nothing	42.2%	54.9%	37.4%
